# Effect of dietary fat intake and genetic risk on glucose and insulin-related traits in Brazilian young adults

**DOI:** 10.1007/s40200-021-00863-7

**Published:** 2021-08-13

**Authors:** Sooad Alsulami, Nathália Teixeira Cruvinel, Nara Rubia da Silva, Ana Carolina Antoneli, Julie A. Lovegrove, Maria Aderuza Horst, Karani Santhanakrishnan Vimaleswaran

**Affiliations:** 1 Department of Food and Nutritional Sciences, Hugh Sinclair Unit of Human Nutrition, University of Reading, Reading, RG6 6DZ UK; 2 Department of Clinical Nutrition, Faculty of Applied Medical Sciences, King Abdulaziz University, Jeddah, Saudi Arabia; 3 Nutritional Genomics Research Group, Faculty of Nutrition, Federal University of Goiás (UFG), Goiania, Goiás, Brazil; 4 Institute for Cardiovascular and Metabolic Research, University of Reading, Reading, UK; 5 Institute for Food, Nutrition, and Health, University of Reading, Reading, UK

**Keywords:** Genetic risk score, Metabolic traits, Brazil, Fat intake, Gene–diet interaction

## Abstract

**Purpose:**

The development of metabolic diseases such as type 2 diabetes (T2D) is closely linked to a complex interplay between genetic and dietary factors. The prevalence of abdominal obesity, hyperinsulinemia, dyslipidaemia, and high blood pressure among Brazilian adolescents is increasing and hence, early lifestyle interventions targeting these factors might be an effective strategy to prevent or slow the progression of T2D.

**Methods:**

We aimed to assess the interaction between dietary and genetic factors on metabolic disease-related traits in 200 healthy Brazilian young adults. Dietary intake was assessed using 3-day food records. Ten metabolic disease-related single nucleotide polymorphisms (SNPs) were used to construct a metabolic-genetic risk score (metabolic-GRS).

**Results:**

We found significant interactions between the metabolic-GRS and total fat intake on fasting insulin level (P_interaction_ = 0.017), insulin-glucose ratio (P_interaction_ = 0.010) and HOMA-B (P_interaction_ = 0.002), respectively, in addition to a borderline GRS-fat intake interaction on HOMA-IR (P_interaction_ = 0.051). Within the high-fat intake category [37.98 ± 3.39% of total energy intake (TEI)], individuals with ≥ 5 risk alleles had increased fasting insulin level (P = 0.021), insulin-glucose ratio (P = 0.010), HOMA-B (P = 0.001) and HOMA-IR (P = 0.053) than those with < 5 risk alleles.

**Conclusion:**

Our study has demonstrated a novel GRS-fat intake interaction in young Brazilian adults, where individuals with higher genetic risk and fat intake had increased glucose and insulin-related traits than those with lower genetic risk. Large intervention and follow-up studies with an objective assessment of dietary factors are needed to confirm our findings.

**Supplementary Information:**

The online version contains supplementary material available at 10.1007/s40200-021-00863-7

## Introduction

Metabolic diseases, such as type 2 diabetes (T2D), have been recognised as a significant public health problem worldwide [1, 2], playing a critical role in medical impoverishment [3–6]. T2D is a major contributor to morbidity and mortality and individuals with T2D have a five-fold increased risk of developing cardiovascular diseases (CVD) [7]**.** The prevalence of diabetes has increased globally (over 463 million adults) [8] but at a faster rate in low- and middle-income countries (LMICs) [9]. In Brazil, the prevalence of T2D has increased by 24% between 2006 and 2019 [10] and an estimate of 65,581 deaths have been shown to be caused by diabetes among adults aged 35–80 years [11]. It has been reported that the prevalence of prediabetes and T2D among Brazilian adolescents were 22.0% and 3.3%, respectively [12]. Studies have also demonstrated the occurrence of cardiometabolic risk factors including abdominal obesity, high insulin levels, dyslipidaemia, and high blood pressure among Brazilian adolescents [12–14]. Hence, early interventions targeting these factors might be an effective strategy to prevent or slow the progression of T2D and decrease the risk of CVD and associated premature mortality [8]**.**

Much of the increase in the prevalence of metabolic diseases in Brazil is attributed to an epidemiological transition characterised by changes in Brazilian age structure, population ageing, reduced rates of infant mortality and fertility and increased low birth weight [15–19]. Changes in the cultural and socioeconomic patterns, for instance, increasing urbanisation and economic improvement, have led to negative changes in lifestyle behaviours, including physical inactivity and unhealthy diet, in the Brazilian adolescent/ young adult population [20]. A previous study has shown that the intake of saturated fatty acids (SFA) was higher in adolescents than adults in Brazil [21]. Animal and human studies have demonstrated an association between increased dietary fat intake and increased insulin resistance [22–24]. In addition, the dietary behaviours of Brazilian young adults have been shown to be characterised by higher intakes of unhealthy foods than middle-aged and older adults, highlighting the need for age-specific public health interventions [25].

The development of metabolic diseases such as T2D is closely linked to a complex interplay between genetic and lifestyle factors, such as diet. Numerous genetic loci have been shown to be associated with T2D [26–29] and related traits [30, 31] and, to date, 243 genetic loci have been identified to be associated with the risk of T2D in multiple ethnic groups [26–29]. Single nucleotide polymorphisms (SNPs) have only a modest effect on disease risk, thus, generating a genetic risk score (GRS) combining several SNPs across the genome is necessary for increasing power to identify disease predisposition patterns of an individual [32]. Evidence has suggested that the genetic risk of metabolic diseases can be modified by dietary intake [33–37]. There are a few gene-diet interaction studies in Brazilians; however, the studies have focused only on cardiovascular disease related traits [38–40]. To date, there are no GRS-diet interaction studies on metabolic traits in Brazilians. Hence, we aimed to investigate the interaction of 10 metabolic disease-related SNPs, as a GRS, with dietary intake on metabolic traits in 200 healthy Brazilian young adults.

## Methodology

### Study population

**Fig. 1:**
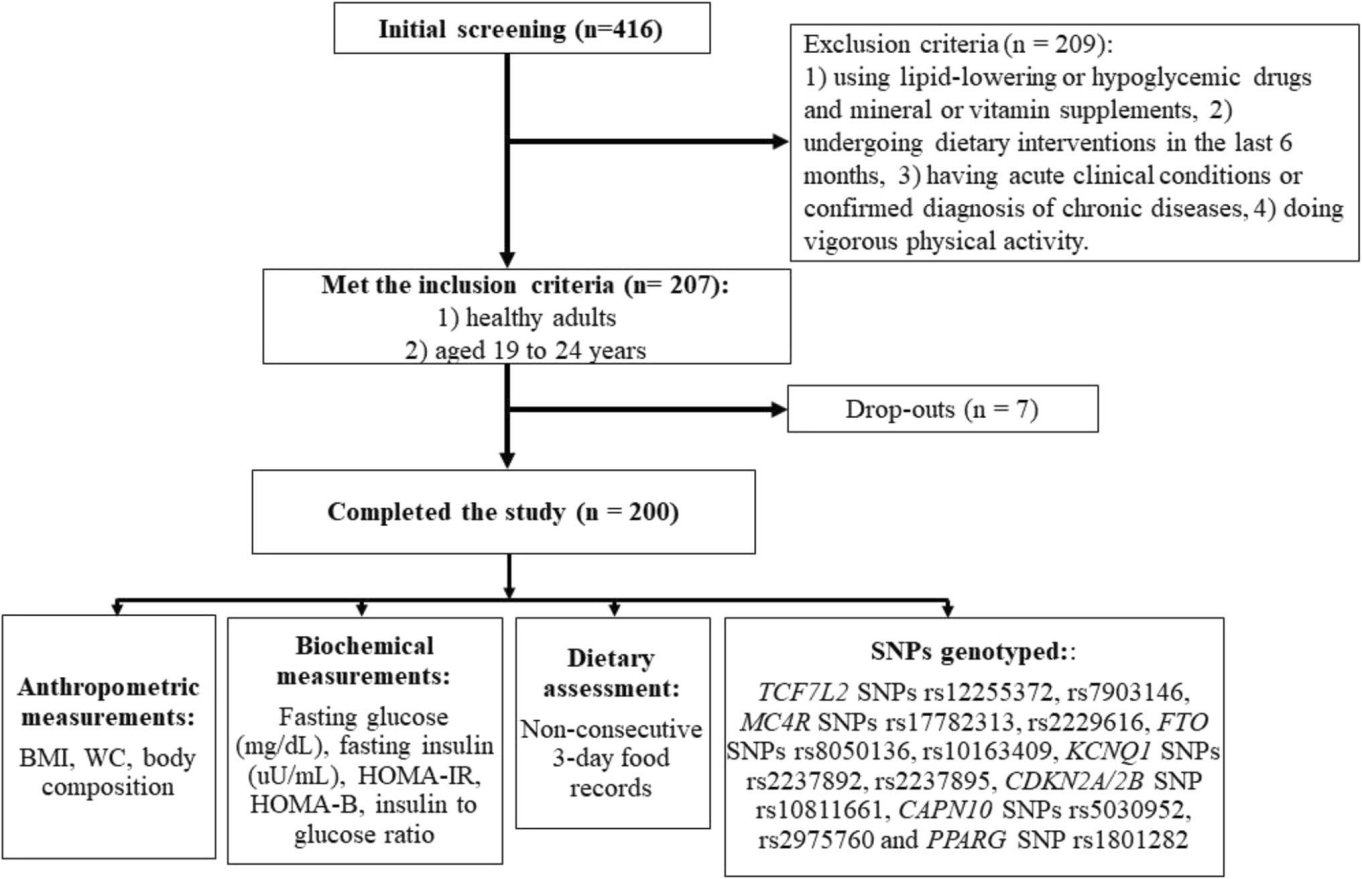
Flow chart showing the participant recruitment process in the BOLD study. In total, 416 individuals were initially screened. After excluding participants based on the exclusion criteria, 207 were included in the study. However, only 200 completed the study. *BMI* body mass index, *WC* waist circumference, *HbA1c* glycated haemoglobin A1c, *HOMA-IR* homeostasis model assessment estimate of insulin resistance, *HOMA-B* homeostasis model assessment estimate of insulin secretion, *TCF7L2* Transcription factor 7-like 2, *MC4R* melanocortin 4 Receptor, *PPARG* Peroxisome proliferator-activated receptor gamma, *FTO* fat mass and obesity-associated, *CDKN2A/2B* Cyclin dependent kinase inhibitor 2A/2B, *KCNQ1* Potassium voltage-gated channel subfamily Q member 1 and *CAPN10* Calpain 10

Obesity, Lifestyle and Diabetes in Brazil (BOLD) is a cross-sectional study of Brazilian healthy young adults aged 19–24 years recruited at the Federal University of Goiás (UFG) between March and June 2019. This study was conducted as part of the ongoing GeNuIne (gene-nutrient interactions) Collaboration, which aims to investigate the impact of genes and lifestyle factors on chronic diseases using data from multiple ethnic groups [41, 42]. All participants completed baseline questionnaires regarding health status, demographics, and socioeconomic status. The study exclusion criteria included those who are 1) using lipid-lowering or hypoglycemic drugs and mineral or vitamin supplements, 2) undergoing dietary interventions in the last 6 months, 3) having acute clinical conditions such as infection, inflammation, fever or diarrhoea, or confirmed diagnosis of chronic diseases such as diabetes mellitus, moderate/severe hypertension, cancer, rheumatoid arthritis and cardiovascular complications, 4) doing vigorous physical activity. In total, 416 individuals showed interest in participating in the study. However, 207 participants met the inclusion criteria and only 200 completed the study (**Fig. ****1****)**. Out of the 200 participants, only 194 had information on genetic and phenotypic measurements as DNA samples were not available for 6 participants. The study was approved by the Ethics Committee of the Federal University of Goiás (protocol number 3.007.456, 08/11/2018), and performed according to the ethical principles in the Declaration of Helsinki. All participants gave written informed consent for study participation.

### Anthropometric and biochemical measurements

Body weight, height and waist circumference (WC) were measured using standardized methods. The weighing was performed on a Tanita® portable electronic scale, with a maximum capacity of 150 kg. For height, a stadiometer with a movable rod was used. WC was measured using an inelastic measuring tape. Body mass index (BMI) was calculated as weight in kilograms divided by height in meters^2^ and WC measurement was taken using a non-extensible measuring tape with partici-pants in light clothing [43]. Body composition was performed by Dual Energy Radiological Absorptiometry (DXA), using the Lunar DPX NT model (General Electric Medical Systems Lunar®; Madison, USA).

Blood samples were collected by peripheral venous puncture in the morning after a 12-h fast and the volunteers were advised not to consume alcohol 72 h before the blood collection. Samples were immediately processed after the collection at the Romulo Rocha Laboratory (Goiânia, Brazil). Fasting serum glucose and insulin were collected in BD Vacutainer® tube and determined by the enzymatic colorimetric method, with an automatic System Vitros Chemistry 950 XRL (Johnson & Johnson, New Brunswick, NJ, USA). Plasma glycated haemoglobin (HbA1c) was collected in an ethylene-diamine-tetra-acetic acid (EDTA) tubes BD Vacutainer® and measured using high-pressure chromatography (HPLC-Bio-Rad Laboratories, Hercules, CA, USA). Plasma samples were obtained by centrifugation at 3500 rpm for ten minutes at 4 °C. The homeostasis model assessment (HOMA) was used to assess the degree of insulin resistance (IR) (HOMA-IR) and β-cell function (HOMA-B). HOMA-IR and HOMA-B were calculated as follows: [fasting insulin levels (mU/l) × fasting glucose levels (mmol/l)/22.5] and [20 × fasting insulin levels)/(fasting glucose levels − 3.5], respectively [44].

### Dietary assessment

Food intake was assessed by trained nutritionists using non-consecutive 3-day food records, including a weekend day [45]. Individuals were provided with measuring cups and spoons of different sizes to assist them in estimating portion size for each food. Foods consumed were converted into grams using the Avanutri Online® diet calculation software (Avanutri Informática Ltda, Rio de Janeiro, Brazil).

### Genotyping

The blood samples (3 ml each) were collected in an EDTA tubes BD Vacutainer® tubes and transported at a controlled temperature (- 80ºC) by the World Courier Company to perform genotyping at LGC Genomics (http://www.lgcgroup.com/services/genotyping), employing the competitive allele-specific PCR-KASP® assay.

### SNP selection and GRS calculation

We selected 12 SNPs that have shown associations with metabolic traits in multiple ethnic groups [26–31]. The detailed information of these SNPs is shown in Table S1. Two SNPs were excluded from the current analysis, as the Calpain 10 (*CAPN10*) rs2975760 SNP was not in Hardy–Weinberg equilibrium (HWE) and the melanocortin 4 Receptor (*MC4R*) rs2229616 SNP had a minor allele frequency (MAF) of less than 1%. Unweighted metabolic-GRS was calculated by summing the number of risk alleles present in each individual. The GRS was generated from the following SNPs: rs12255372, rs7903146 of the Transcription factor 7-like 2 (*TCF7L2*) gene, rs17782313 of the *MC4R* gene, rs8050136 and rs10163409 of the fat mass and obesity-associated (*FTO*), rs2237892 and rs2237895 of the Potassium voltage-gated channel subfamily Q member 1(*KCNQ1*) gene, rs10811661 of the Cyclin dependent kinase inhibitor 2A/2B (*CDKN2A/2B*) gene, rs5030952 of the *CAPN10* gene, and rs1801282 of the Peroxisome proliferator-activated receptor gamma (*PPARG*) gene. Genotypes were coded as 0, 1, or 2 according to the number of metabolic-associated risk alleles that are defined based on the literature. These values were then calculated by summing the number of risk alleles for each variant. The GRS was then categorised based on the median risk alleles into two categories: “GRS < 5 risk alleles” and “GRS ≥ 5 risk alleles”.

### Statistical analysis

Descriptive characteristics of the study population stratified by sex were presented as means and standard deviation (SDs) for continuous variables and compared using an independent samples t-test. Variables were tested for normality using Shapiro–Wilk's W test and non-normally distributed variables were log-transformed including BMI, WC, body fat mass percentage, HbA1c, fasting glucose, fasting insulin, HOMA-IR, HOMA-B, insulin to glucose ratio, total energy intake (TEI), carbohydrate %, protein %, SFA %, and polyunsaturated fatty acids (PUFA) %. We investigated the effects of metabolic-GRS on metabolic traits using general linear models. To test the interactions of the metabolic-GRS with dietary factors on metabolic traits, we included the interaction term (e.g., GRS*fat intake) in the models. The dietary factors investigated in our study included the total dietary intake of fat, protein, and carbohydrate (percentages of TEI). Significant interactions between the GRS and the total fat intake were analysed in more depth to determine the effect of fat subtypes including SFA, monounsaturated fatty acids (MUFA), and PUFA. The GRS-nutrient interactions that reached statistical significance (p < 0.05) were tested for the effects of the GRSs on metabolic traits according to tertiles of dietary intakes (low, medium and high intake) using general linear models. All models were adjusted for age, sex and BMI (when BMI is not an outcome). Given that insulin levels are influenced by both the capacity for insulin secretion and IR [46, 47], analysis of HOMA-B was performed with and without adjustment for IR to improve the accuracy of pancreatic β‐cell function estimate. All statistical tests were two-sided, and analyses were performed using Statistical Package for the Social Sciences (SPSS) software (version 24; SPSS Inc., Chicago, IL, USA).

## Results

### Characteristics of the study participants

Table 1 summarises the characteristics of individuals in this study according to sex. Men had higher BMI, WC, fasting glucose, and lower fat mass % compared to women (P < 0.05 for all). Men also reported higher intakes of total energy and protein than women (P < 0.05 for all).

**Table 1:** Characteristics of study participants

Parameters	Total (n = 200)	Women (n = 147)	Men (n = 53)	*p*‐Value
Age (years)	21.35 ± 1.67	21.33 ± 1.70	21.40 ± 1.61	0.815
BMI (kg/m2)	23.35 ± 4.42	22.81 ± 3.97	24.86 ± 5.23	**0.004**
WC (cm)	74.55 ± 13.56	71.10 ± 12.05	84.13 ± 13.01	**0.000**
Body fat mass (%)	33.91 ± 10.72	37.17 ± 8.77	24.84 ± 10.48	**0.000**
HbA1c (%)	4.73 ± 0.25	4.71 ± 0.25	4.78 ± 0.26	0.103
Fasting serum glucose (mg/dL)	87.18 ± 6.84	86.43 ± 6.78	89.26 ± 6.60	**0.009**
Fasting serum insulin (uU/mL)	8.74 ± 3.80	8.69 ± 3.37	8.88 ± 4.82	0.784
HOMA-IR	1.89 ± 0.88	1.86 ± 0.76	1.98 ± 1.15	0.513
HOMA-B	138.32 ± 65.75	142.47 ± 65.65	126.81 ± 65.25	0.137
Insulin to glucose ratio	0.10 ± 0.04	0.10 ± 0.04	0.10 ± 0.05	0.944
Energy (Kcal/day)	1827.81 ± 597.94	1741.52 ± 558.82	2067.15 ± 641.91	**0.001**
Protein (energy %)	17.11 ± 3.63	16.74 ± 3.33	18.14 ± 4.24	**0.016**
Carbohydrate (energy %)	51.09 ± 7.11	51.11 ± 7.01	51.05 ± 7.44	0.961
Fat (energy %)	31.66 ± 5.83	32.12 ± 5.69	30.38 ± 6.08	0.061
SFA (%)	9.43 ± 5.43	9.54 ± 6.030	9.14 ± 3.25	0.652
PUFA (%)	5.13 ± 2.27	5.08 ± 2.38	5.26 ± 1.92	0.628
MUFA (%)	7.72 ± 2.63	7.55 ± 2.55	8.19 ± 2.79	0.129

Data presented as the mean ± SDs. *P* values for the differences in the means between men and women were calculated using the independent samples t-test. *BMI* body mass index, *WC* waist circumference, *HbA1c* glycated haemoglobin, *HOMA-IR* homeostasis model assessment estimate of insulin resistance, *HOMA-B* homeostasis model assessment estimate of insulin secretion, *SFA* saturated fatty acids, *MUFA* monounsaturated fatty acids, *PUFA* polyunsaturated fatty acids

### Associations between metabolic-GRS and metabolic traits

None of the associations between metabolic-GRS and metabolic-disease related traits was statistically significant except for the association with BMI (P = 0.008) (Table 2).

**Table 2:** Associations of metabolic-GRS with metabolic traits

Parameters	GRS < 5 (n = 93)	GRS ≥ 5 (n = 101)	*p*‐Value
BMI (kg/m2)	23.90 ± 0.43	22.60 ± 0.43	**0.008**
WC (cm)	75.53 ± 1.27	73.93 ± 1.26	0.967
Body fat mass (%)	35.80 ± 1.05	31.91 ± 1.10	0.663
HbA1c (%)	4.72 ± 0.03	4.73 ± 0.03	0.964
Fasting glucose (mg/dL)	87.54 ± 0.68	86.74 ± 0.72	0.419
Fasting insulin (uU/mL)	8.91 ± 0.43	8.52 ± 0.34	0.542
HOMA-IR	1.93 ± 0.10	1.84 ± 0.08	0.663
HOMA-B	138.76 ± 7.15	138.17 ± 6.32	0.234
HOMA-B adjusted for HOMA-IR	138.76 ± 7.15	138.17 ± 6.32	0.235
Insulin to glucose ratio	0.10 ± 0.00	0.10 ± 0.00	0.477

Data are Mean ± standard error of the mean (SEM). *P* values obtained from the linear regression analysis adjusted for age, sex and additionally for BMI when BMI is not an outcome. The analysis was performed on log-transformed variables. *GRS* genetic risk score, *BMI* body mass index, *WC* waist circumference, *HbAIc* glycated haemoglobin, *HOMA-IR* homeostasis model assessment estimate of insulin resistance, *HOMA-B* homeostasis model assessment estimate of insulin secretion

### Interactions of metabolic-GRS with dietary factors on metabolic traits

**Fig. 2:**
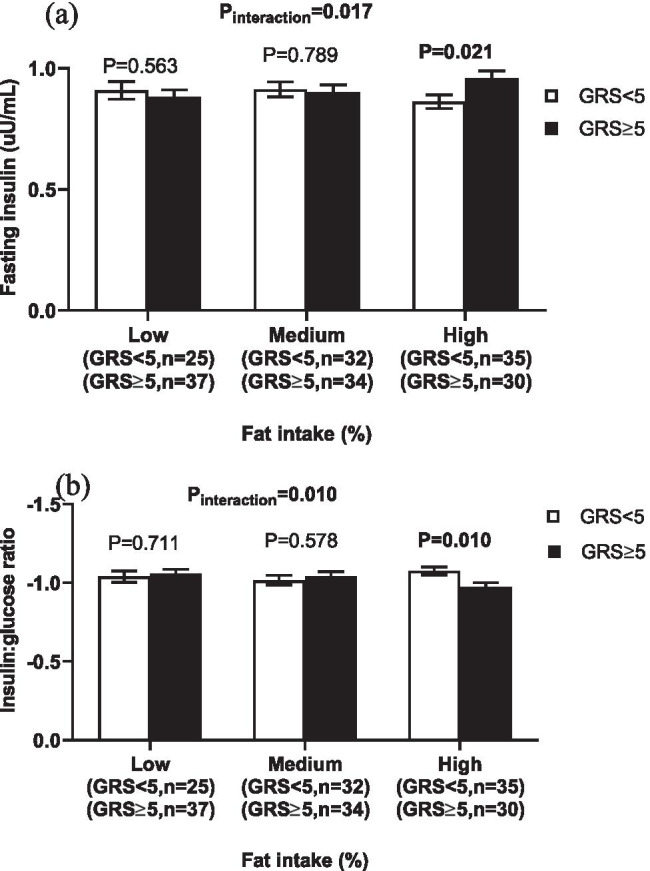
Interaction between the metabolic-GRS and fat intake (%) on fasting insulin levels and insulin: glucose ratio. White bars indicate individuals with GRS < 5 risk alleles; the black bars indicate individuals with GRS ≥ 5 risk alleles; Error bars indicate the standard error of the mean. Individuals with ≥ 5 risk alleles had higher fasting insulin (**a**) and insulin to glucose ratio (**b**) compared to those with < 5 risk alleles, among individuals with a higher total fat intake (37.98 ± 3.39% of TEI). *GRS* genetic risk score, *TEI* total energy intake

**Fig. 3:**
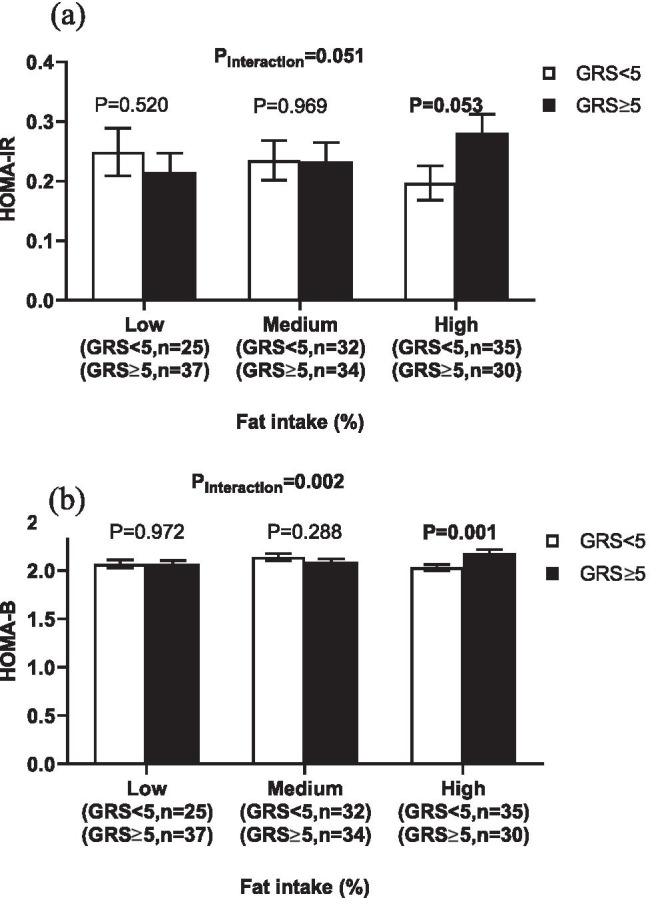
Interaction between the metabolic-GRS and fat intake (%) on HOMA-IR and HOMA-B. White bars indicate individuals with GRS < 5 risk alleles; the black bars indicate individuals with GRS ≥ 5 risk alleles; Error bars indicate the standard error of the mean. Individuals with ≥ 5 risk alleles had higher HOMA-IR (**a**) and HOMA-B (**b**) compared to those with < 5 risk alleles, among individuals with a higher total fat intake (37.98 ± 3.39% of TEI). *GRS* genetic risk score, *TEI* total energy intake, *HOMA‐IR* homeostasis model assessment estimate of insulin resistance, *HOMA‐B* homeostasis model assessment estimate of insulin secretion

As shown in Table 3, there were statistically significant interactions between the metabolic-GRS and total fat intake (% of TEI) on fasting insulin level (P_interaction_ = 0.017), insulin-glucose ratio (P_interaction_ = 0.010) and HOMA-B (P_interaction_ = 0.002) and a borderline interaction on HOMA-IR (P_interaction_ = 0.051). Among those in the highest tertile of fat intake (37.98 ± 3.39% of TEI), individuals with ≥ 5 risk alleles had higher fasting insulin level (P = 0.021), insulin-glucose ratio (P = 0.010), HOMA-B (P = 0.001) and HOMA-IR (P = 0.053), compared to those with < 5 risk alleles (Figs. 2 and 3). Interaction on HOMA-B was still significant after adjusting the analysis for HOMA-IR (P_interaction_ = 0.016), Figure S1. We further examined interactions with fat subtypes on these traits. Significant interactions were detected between the metabolic-GRS and MUFA intake on fasting insulin (P_interaction_ = 0.021), HOMA-IR (P_interaction_ = 0.021) and insulin to glucose ratio (P_interaction_ = 0.031), however, none of these interactions was statistically significant after tertile analysis. Significant interactions were also observed between the metabolic-GRS and intakes of total fat, PUFA and MUFA on percentage of body fat mass (P_interaction_ = 0.009, 0.033 and 0.038, respectively).

**Table 3:** Interactions of the metabolic-GRS with dietary factors on metabolic traits

	Protein (%)	Carbohydrate (%)	Fat (%)	SFA (%)	PUFA (%)	MUFA (%)
BMI (kg/m2)	0.255	0.120	0.922	-	-	-
WC (cm)	0.124	0.303	0.979	-	-	-
Body fat mass (%)	0.451	0.311	**0.009**	0.255	**0.033**	**0.038**
HbA1c (%)	0.955	0.653	0.632	-	-	-
Fasting glucose (mg/dL)	0.764	0.142	0.099	-	-	-
Fasting insulin (uU/mL)	0.898	0.37	**0.017**	0.233	0.809	**0.021**
HOMA-IR	0.944	0.561	**0.051**	0.357	0.837	**0.021**
HOMA-B	0.797	0.089	**0.002**	0.079	0.749	0.123
HOMA-B adjusted for HOMA-IR	0.784	0.084	**0.016**	0.131	0.806	0.952
Insulin to glucose ratio	0.895	0.274	**0.010**	0.154	0.801	**0.031**

Data are P values of interaction which obtained from the linear regression analysis adjusted for age, sex and additionally for BMI when BMI is not an outcome. The analysis was performed on log-transformed variables. *GRS* genetic risk score, *BMI* body mass index, *WC* waist circumference, *HbA1c* glycated haemoglobin, *HOMA-IR* homeostasis model assessment estimate of insulin resistance, *HOMA-B* homeostasis model assessment estimate of insulin secretion, *SFA* saturated fatty acids, *MUFA* monounsaturated fatty acids, *PUFA* polyunsaturated fatty acids

## Discussion

The present study investigated the potential interplay between metabolic-GRS and dietary macronutrient intake on metabolic traits in a Brazilian young adult population. Our results provide evidence of significant GRS-fat intake interactions on glucose and insulin-related traits, where individuals with ≥ 5 risk alleles had higher fasting insulin level, insulin-glucose ratio, HOMA-IR and HOMA-B than those with < 5 risk alleles among those in the high fat intake group (37.98 ± 3.39% of TEI). These findings suggest that individuals with ≥ 5 risk alleles are sensitive to dietary fat with respect to glucose and insulin-related traits and that these individuals may derive the most benefit from following the Brazilian dietary guidelines which aim at reducing fat intake to less than 30% of TEI [48]. This could have significant implication for public health in terms of providing early intervention to young adults with high genetic risk before the onset of disease, which might help halt the development of T2D.

In the present study, the metabolic-GRS was found to be associated with lower BMI, which contradicts the findings of the previous GRS-related studies in European populations [49–52]. However, the Brazilian population has a mixed genetic ancestry that originates from Europeans, Africans and Native Amerindians, which might explain the discrepancies between our findings and genetic association studies in Europeans [53]. Furthermore, a large GWAS of 241,258 European adults showed that the risk allele T of *TCF7L2* rs7903146 was associated with lower BMI compared to the non-risk allele, which may provide a possible explanation of our findings [54]. Metabolic diseases are complex and multifactorial influenced by both environmental and genetic factors including dozens or even hundreds of genetic variants each contributing small effects on these traits [55, 56]. Thus, the effect of unmeasured factors on BMI might influence the observed findings.

The present study found that, within the high-fat intake category, individuals with higher metabolic-GRS showed increased fasting insulin level, insulin-glucose ratio, HOMA-IR and HOMA-B, whereas those with lower GRS showed a reduction in these traits. Although direct comparison of our study with previous gene-diet interaction studies is difficult due to differences in the methodology related to the construction of GRSs and measurement of dietary intake, sample size, study design, and ethnicity, our findings are in agreement with some of the previous studies in other populations in which fat intake was found to interact with GRS on metabolic traits [33–35]. In a recent study in 302 Ghanaian adults, a GRS of 4 metabolic-related variants was associated with higher WC among individuals with high fat intake (34.99 ± 5.54% TEI) [57]. Data from an intervention study in 733 European adults also reported that higher total fat intake was associated with increased fasting glucose in individuals with higher GRS of 14 fasting glucose-associated SNPs and with decreased fasting glucose among individuals with lower GRS [33]. Taken together, these observations suggest that individuals with higher genetic risk might benefit more from reducing fat intake in terms of lowering their metabolic risk.

Dietary guidelines have recommended to limit the dietary intake of total fat (between 15 and 30% of TEI) to preserve overall health and reduce the risk of developing metabolic diseases [58]. Previous studies have demonstrated that the higher intake of total fat contributes to the development of T2D by inducing IR [24, 59]. Lowering total fat intake have been reported to improve glycemic control in a systematic review of clinical trials of diabetic individuals [60]. Evidence from two previous intervention studies including individuals from various ethnic groups (n = 3,234 and 522, respectively) and with long follow-up (2.8 and 3.2 years, respectively) have also shown that decreasing fat intake (from 6.6 ± 0.2% of TEI and to < 30% of TEI, respectively) is effective in reducing the incidence of T2D by up to 58% [61, 62]. In addition, dietary intervention in 48,835 postmenopausal women from the US showed that reducing total fat intake (by ~ 8% of TEI) and increasing carbohydrate intake (by ~ 8% of TEI) through increasing intake of vegetable/fruit (five servings per day) and grain (six servings per day) were associated with a reduction in glycemia and diabetes progression [63]. The dietary intake of Brazilians is characterised by unfavourable fat profile with high intakes of SFA and trans fatty acids and imbalances in the omega-6:omega-3 ratio, being compatible with a high risk of metabolic diseases [21]. In our study, the mean fat intake of the total sample (31.66 ± 5.8% of TEI) and the high fat intake group (37.98 ± 3.39% of TEI) were above the recommended dietary guidelines for Brazilian adults (< 30% of TEI) [64].

The mechanisms by which dietary fat influences IR and β-cell function are unclear; however, several pathways are biologically plausible. IR is often mediated by increased inflammation that has been shown to be induced mostly by the effect of the fatty acids composition of the diet [65]. In particular, SFA and omega-6 have pro-inflammatory effects, and omega-3 fatty acids have anti-inflammatory effects [65]. Some of the molecular mechanisms of IR include the lipid-overload hypothesis in which ceramides or diacylglycerides are accumulated leading to the inhibition of insulin signalling and oxidative stress induced by excessive generation of free radicals or endoplasmic reticulum stress induced by excessive calorie intake [66–68]. In addition to the insulin-related traits, there was also a significant interaction between GRS and intakes of total fat, PUFA and MUFA on the percentage of body fat mass in our study. Given that adipose tissue is a central metabolic organ that stores excess fat energy in the form of lipid and secretes proinflammatory adipokines that can also influence signalling of insulin, our finding is biologically plausible [69]. It is worth observing the intake of SFA, PUFA and MUFA which were significantly higher in the high fat intake category than low and medium intake groups; this might be one of the reasons for the observed interactions with total dietary fat intake. Evidence suggests that different types of dietary fat have differential effects on IR and insulin secretion. While a cross-sectional study in 538 Spanish individuals suggested that the intake of a MUFA-rich diet was associated with increased HOMA-B [70], a meta-analysis of randomised controlled feeding trials (n = 4220) demonstrated that PUFA intake showed the most consistent favourable effects in relation to improved glycaemia and insulin secretion capacity [71].

Several strengths are worth consideration. This study is the first to examine whether dietary factors interact with metabolic-GRSs on metabolic traits among the Brazilian young adult population. Early prediction of insulin sensitivity in young adults and effective intervention can be a critical factor in terms of delaying or preventing diabetes in normoglycemic individuals who are at risk of diabetes [72]. Also, a GRS analysis approach was used, which has the advantage over single-locus approach [32]. This approach is especially important for highly polygenic metabolic traits and can identify individuals at risk of metabolic diseases who might benefit from targeted interventions [32]. Furthermore, the study outcomes (metabolic traits) were measured using validated methods by trained staff which improve the accuracy of these estimates. Nevertheless, some limitations need to be acknowledged. A major limitation is the small sample size, suggesting that our analysis might be underpowered. However, the use of the GRS approach is suggested to improve the power and significant gene-diet interactions were detected in our study. As with all observational studies, causality between exposure and outcome cannot be inferred and residual confounders might have existed. Given the longitudinal dimension of the development of T2D and the complexity of gene-diet interactions, our cross-sectional study design fails to determine the temporality of the observed findings. Given that dietary intake was assessed using self-reported measures, we cannot exclude the effect of measurement bias. Another limitation is that the effect of different dietary sources of fat (including meat, dairy and plant) were not considered in the present analysis, which might have provided further explanations to our GRS-fat intake interactions [73]. In addition, our GRS was constructed based on 10 SNPs, which account for only a small proportion of the metabolic disease-related genetic variants. As HOMA is a widely validated clinical and epidemiological tool for assessing IR and β-cell function [74], like many other epidemiological studies [33, 35, 59], we also used HOMA-IR and HOMA-B as proxies for IR and insulin secretion, respectively. However, these measures are calculated only using fasting insulin and glucose values which might provide different estimates compared to methods based on dynamic measurements of insulin and glucose responses or those derived from clamp experiments [75]. Finally, given that the study was performed with relatively healthy overweight/obese young individuals with normal glucose tolerance who might have a quicker adaptation to changes in fat intake, the findings might not be applicable to those with impaired glucose metabolism or diabetes.

In conclusion, our study provides evidence of interactions between genetic predisposition and high fat intake on diabetes-related traits among Brazilian young adults. These findings encourage identifying Brazilian young adults with high genetic risk and tailoring dietary recommendations of fat intake according to their metabolic genetic risk profile for the primary prevention of adult-onset T2D. In addition, devising polygenic risk score could be used to provide more insights on understanding the pathophysiology of the genetics of diabetes. However, large interventional and follow up studies with a more comprehensive and objective assessment of environmental factors are needed in Brazilians to confirm our findings and to evaluate the clinical benefit of implementing precision dietary interventions based on an individual’s underlying genetic risk of metabolic diseases.

## Supplementary Information

Below is the link to the electronic supplementary material.

Supplementary file1 (DOCX 42 KB)

## Data Availability

The data that support the findings of this study are available from the corresponding author (KSV) upon reasonable request.
