# Supplementary material for: Effect of dietary fat intake and genetic risk on glucose and insulin-related traits in Brazilian young adults
Source: J Diabetes Metab Disord. 2021 Aug 13;20(2):1337–47. doi: 10.1007/s40200-021-00863-7 (PMC8630327; doi:10.1007/s40200-021-00863-7)
Supplement: Supplementary file 1 — Supplementary file1 (DOCX 42 KB) [file 40200_2021_863_MOESM1_ESM.docx]

**Supplementary file**

**Table S1.** Genotype distribution of the twelve SNPs that were chosen for our study.

| **Gene name** | **SNP** | **Location of the SNP** | **Genotype** | | **Minor Allele** | **MAF** | **HWE** |
| --- | --- | --- | --- | --- | --- | --- | --- |
| Transcription factor 7-like 2 *(TCF7L2)* | rs12255372 | Intron Variant | GG | 85 | T | 0.35 | 0.80 |
|  |  |  | TG | 92 |  |  |  |
|  |  |  | TT | 23 |  |  |  |
| Transcription factor 7-like 2 *(TCF7L2)* | rs7903146 | Intron Variant | CC | 90 | T | 0.33 | 0.78 |
|  |  |  | TC | 87 |  |  |  |
|  |  |  | TT | 23 |  |  |  |
| Melanocortin 4 Receptor *(MC4R)* | rs17782313 | - | CC | 6 | C | 0.17 | 0.84 |
|  |  |  | TC | 55 |  |  |  |
|  |  |  | TT | 139 |  |  |  |
| Melanocortin 4 Receptor *(MC4R)* | rs2229616 | Missense Variant | AA | 0 | A | 0.00 | 0.97 |
|  |  |  | GA | 1 |  |  |  |
|  |  |  | GG | 199 |  |  |  |
| Peroxisome proliferator-activated receptor gamma *(PPARG)* | rs1801282 | Missense Variant | CC | 170 | G | 0.08 | 0.25 |
|  |  |  | GC | 30 |  |  |  |
|  |  |  | GG | 0 |  |  |  |
| Fat mass and obesity-associated *(FTO)* | rs8050136 | Intron Variant | AA | 30 | A | 0.40 | 0.79 |
|  |  |  | CA | 96 |  |  |  |
|  |  |  | CC | 71 |  |  |  |
| Fat mass and obesity-associated *(FTO)* | rs10163409 | Intron Variant | AA | 117 | T | 0.25 | 0.06 |
|  |  |  | TA | 64 |  |  |  |
|  |  |  | TT | 17 |  |  |  |
| Cyclin dependent kinase inhibitor 2A/2B *(CDKN2B)* | rs10811661 | - | CC | 4 | C | 0.13 | 0.76 |
|  |  |  | CT | 45 |  |  |  |
|  |  |  | TT | 151 |  |  |  |
| Potassium voltage-gated channel subfamily Q member *(KCNQ1)* | rs2237895 | Intron Variant | AA | 85 | C | 0.34 | 0.86 |
|  |  |  | CA | 91 |  |  |  |
|  |  |  | CC | 23 |  |  |  |
| Potassium voltage-gated channel subfamily Q member *(KCNQ1)* | rs2237892 | Intron Variant | CC | 160 | T | 0.11 | 0.08 |
|  |  |  | TC | 35 |  |  |  |
|  |  |  | TT | 5 |  |  |  |
| Calpain 10 *(CAPN10)* | rs2975760 | Intron Variant | CC | 15 | C | 0.15 | <0.0001 |
|  |  |  | TC | 31 |  |  |  |
|  |  |  | TT | 154 |  |  |  |
| Calpain 10 *(CAPN10)* | rs5030952 | - | CC | 128 | T | 0.20 | 0.47 |
|  |  |  | TC | 66 |  |  |  |
|  |  |  | TT | 6 |  |  |  |

Abbreviations: SNP, single nucleotide polymorphisms; GRS, genetic risk score; MAF, minor allele frequency; HWE, Hardy-Weinberg equilibrium; *TCF7L2*, Transcription factor 7-like 2; *MC4R*, melanocortin 4 Receptor; *PPARG*, Peroxisome proliferator-activated receptor gamma; *FTO*, fat mass and obesity-associated; *CDKN2A/2B*, Cyclin dependent kinase inhibitor 2A/2B; *KCNQ1*, Potassium voltage-gated channel subfamily Q member 1; *CAPN10*, Calpain 10

**Figure S1.** Interaction between the metabolic-GRS and fat intake (%) on HOMA-B after adjustment of HOMA-IR. White bars indicate individuals with GRS <5 risk alleles; the black bars indicate individuals with GRS ≥5 risk alleles; Error bars indicate the standard error of the mean. Individuals with ≥5 risk alleles had higher HOMA-B compared to those with<5 risk alleles, among individuals with a higher total fat intake (37.98±3.39 % of TEI). Abbreviations: GRS: genetic risk score; TEI: total energy intake; HOMA‐IR: homeostasis model assessment estimate of insulin resistance; HOMA‐B: homeostasis model assessment estimate of insulin secretion.
